# A Nonlethal Murine Flame Burn Model Leads to a Transient Reduction in Host Defenses and Enhanced Susceptibility to Lethal Pseudomonas aeruginosa Infection

**DOI:** 10.1128/IAI.00091-21

**Published:** 2021-09-16

**Authors:** Jerod Brammer, Myeongjin Choi, Scott M. Baliban, Adrienne R. Kambouris, Gary Fiskum, Wei Chao, Kerri Lopez, Catriona Miller, Yousef Al-Abed, Stefanie N. Vogel, Raphael Simon, Alan S. Cross

**Affiliations:** a Center for Vaccine Development and Global Health, University of Maryland School of Medicine, Baltimore, Maryland, USA; b Department of Microbiology and Immunology, University of Maryland School of Medicine, Baltimore, Maryland, USA; c Translational Research Program, Department of Anesthesiology & Center for Shock, Trauma and Anesthesiology Research, University of Maryland School of Medicine, Baltimore, Maryland, USA; d Department of Surgery, University of Maryland School of Medicine, Baltimore, Maryland, USA; e Enroute Care Division, Department of Aeromedical Research, USAF School of Aerospace Medicine, Wright Patterson AFB, Dayton, Ohio, USA; f Department of Biomedical Science, The Feinstein Institute for Medical Research, Manhasset, New York, USA; Stanford University

**Keywords:** flame burn, HMGB1, murine, P5779, *Pseudomonas aeruginosa*, sepsis

## Abstract

Of the 486,000 burn injuries that required medical treatment in the United States in 2016, 40,000 people were hospitalized, with >3,000 fatalities. After burn injury, humans are at increased risk of sepsis and mortality from infections caused by Pseudomonas aeruginosa, an opportunistic pathogen. We hypothesize that systemic events were initiated from the burn that increased the host’s susceptibility to P. aeruginosa. A nonlethal 10% total body surface area (TBSA), full-thickness flame burn was performed in CD-1 mice without and with subsequent P. aeruginosa (strain M2) infection. The 50% lethal dose for subcutaneous infection with P. aeruginosa M2 at the burn site immediately after the burn decreased by 6 log, with mortality occurring between 18 and 26 h, compared with P. aeruginosa-infected mice without burn injury. Bacteria in distal organs were detected by 18 h, concurrent with the onset of clinical symptoms. Serum proinflammatory cytokines (interleukin-6 [IL-6], IL-1β, gamma interferon, and tumor necrosis factor alpha) and the anti-inflammatory cytokine IL-10 were first detected at 12 h postburn with infection and continued to increase until death. Directly after burn alone, serum levels of HMGB1, a danger-associated molecular pattern and TLR4 agonist, transiently increased to 50 ng/ml before returning to 20 ng/ml. Burn with P. aeruginosa infection increased serum HMGB1 concentrations >10-fold (250 ng/ml) at the time of death. This HMGB1-rich serum stimulated TLR4-mediated NF-κB activation in a TLR4 reporter cell line. Treatment of infected burned mice with P5779, a peptide inhibitor of HMGB1, increased the mean survival from 23 to 42 h (*P* < 0.0001). We conclude that the high level of serum HMGB1, which preceded the increase in proinflammatory cytokines, is associated with postburn mortality.

## INTRODUCTION

The American Burn Association reported 486,000 burn injuries that required medical treatment in 2016 and that 40,000 of these injuries led to hospitalization. Forty-three percent of these burns were related to flames, with 17,000 burn center admissions and >3,000 deaths. The United States continues to see widespread wildfires that are likely to expose emergency responders and the public to flame-based burns. Burn size and depth are determined by the amount and duration of heat transferred to the skin. When the skin reaches 48°C, cellular death occurs and results in a first-degree burn. Once the temperature exceeds 72°C, the tissue is instantly destroyed (1). Burn injuries are classified from first- to third-degree burns, with first-degree burns being superficial, requiring minimal treatment. Third-degree burns, described as full-thickness burns, extend beyond the dermal layers and destroy underlying bone, muscle, and/or tendons, requiring extensive medical intervention that includes skin grafting (2).

Individuals who survive large full-thickness burns are at increased risk of developing bacterial infections that may be the result of contamination of the open burn wound outside a clinical setting or from hospital-acquired infections during treatment (3). These bacterial infections can lead to sepsis, which in burn patients differs from non-burn-induced sepsis in that the primary barrier to infection, the skin, is damaged or lost. This increases the period of exposure to opportunistic pathogens while the wound is open and is a source of continual inflammatory signaling at the wound site (4, 5). Pseudomonas aeruginosa, a ubiquitous Gram-negative bacterium, is one of the most common opportunistic pathogens identified from burn wounds (6). P. aeruginosa does not routinely cause disease in immunocompetent mice or humans. Immunosuppressed patients have a higher likelihood of gastrointestinal colonization with P. aeruginosa both before and after admittance into the hospital, which increases the likelihood of serious postburn infections (7).

Mice have been shown to closely recapitulate immunological transcriptomic responses compared to humans during burn injuries (8). Consequently, mouse burn models are routinely used to assess bacterial dissemination and treatment during postburn sepsis. Some of the more common models for producing full-thickness burns in animals are achieved with hot water baths or heated brass rods (9, 10). These methods are ideal for mimicking burns from scalds or touching hot objects but may not fully compare to a flame-inflicted burn. To mimic the events of flame burns, our laboratory performed an ethanol-based flame burn to achieve a full-thickness, 10% total body surface area (TBSA) nonlethal burn, a model that was originally developed in 1975, which revealed that directly after a full-thickness burn, mice are extremely susceptible to subcutaneous (s.c). infection from P. aeruginosa strain M2 (O5:FlaB) (11). A complete understanding of the immune response to P. aeruginosa colonization, dissemination, and development of sepsis postburn in CD-1 mice has not been fully achieved. Our findings indicate that alterations in host responses contribute significantly to increased susceptibility to P. aeruginosa M2 in CD-1 mice post-10% flame burn. HMGB1, a danger-associated molecular pattern (DAMP) previously associated with sepsis (12) and shown to activate TLR4 signaling (13), was released into serum within minutes after the burn, resulting in an increase in TLR4-mediated proinflammatory signaling. Treating mice with P5779, an HMGB1 antagonist, increased the median time of death and survival postburn following an s.c. infection of P. aeruginosa M2.

## RESULTS

### A flame burn produced a full-thickness burn.

**FIG 1:**
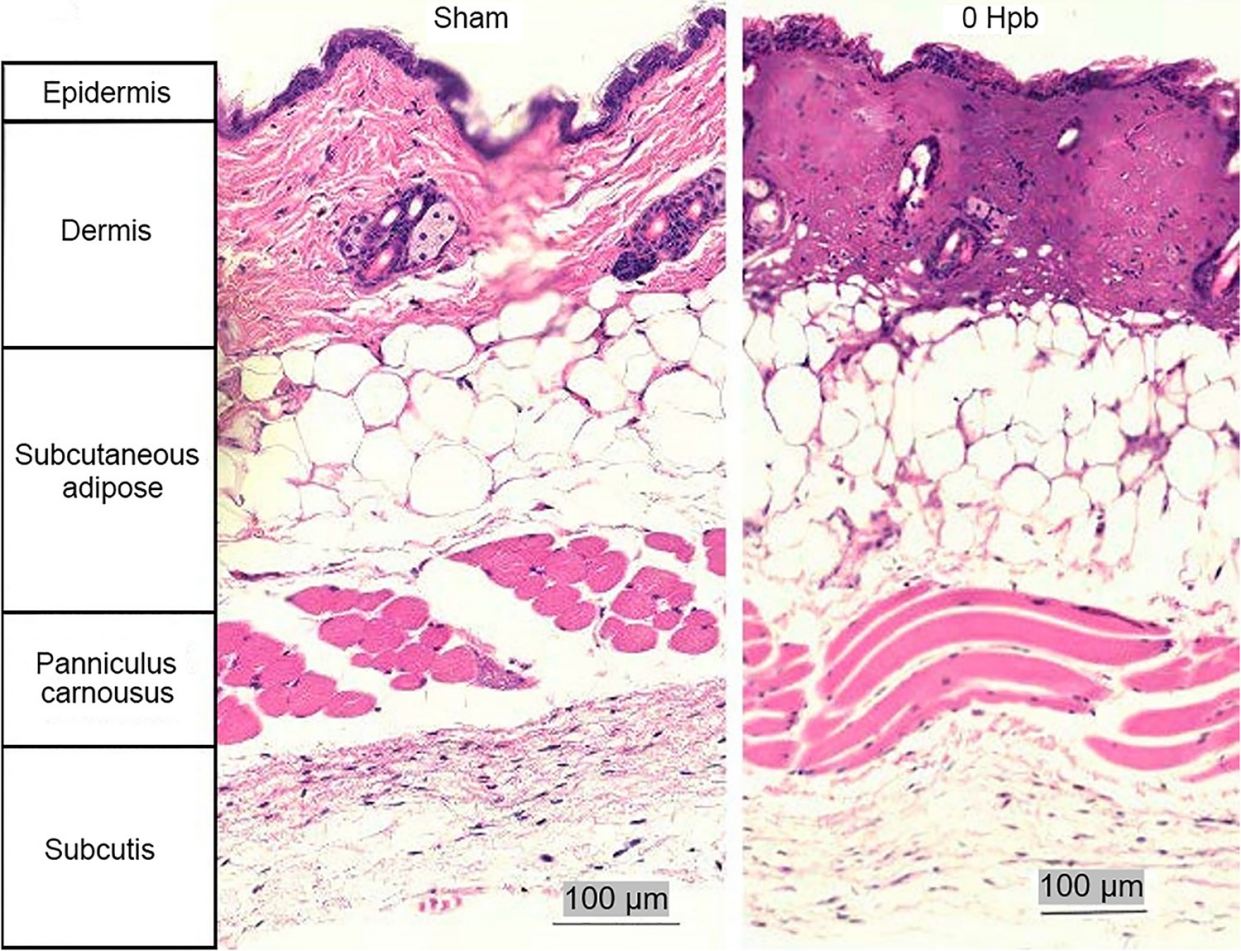
Burn depth using H&E staining of sham and burned skin. Burned skin shows destruction of the epidermis and dermis but sparing of deeper tissues.

Histological analysis of skin by hematoxylin and eosin (H&E) staining at the time of the burn, in the absence of infection, produced a full-thickness burn that extended through the epidermal and dermal layers, with complete loss of cellular structure compared to the skin of sham-treated mice. The cutaneous adipose tissue and the panniculus carnosus were spared from injury (Fig. 1).

### A 10% TBSA burn resulted in increased susceptibility to P. aeruginosa M2 sepsis and bacterial dissemination to the blood and organs.

**FIG 2:**
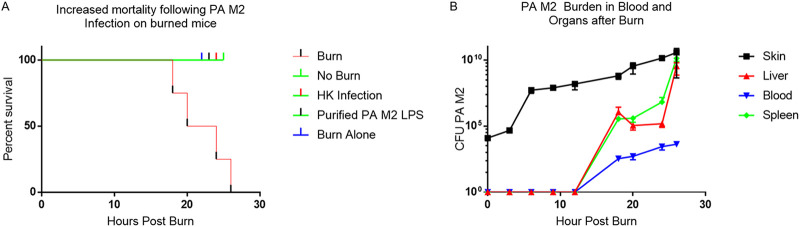
Skin and distal-organ P. aeruginosa (PA) burden and mortality. (A) Kaplan-Meier survival curves in response to burn with P. aeruginosa M2 (*n* = 10), sham with P. aeruginosa M2 (*n* = 6), burn plus HK P. aeruginosa M2 (*n* = 6), burn plus purified P. aeruginosa M2 LPS (*n* = 6), and burn alone (*n* = 6). Mice were observed for morbidity for 7 days. A log-rank test was used to compare survival between groups. ****, *P < *0.0001. (B) Blood and tissue were collected at various times from burn mice that were infected with P. aeruginosa M2 to determine bacterial burden levels. The latest time point achievable was 26 h due to the onset of morbidity.

After receiving a 10% TBSA burn, mice were randomly assigned into five groups, each receiving 100 μl subcutaneously of 3.3 × 10^4^ CFU P. aeruginosa M2, 3.3 × 10^4^ CFU of heat-killed (HK) P. aeruginosa M2, 10 pg of P. aeruginosa M2 lipopolysaccharide (LPS), representative of the LPS content of 10^4^ CFU P. aeruginosa M2, 100 μl phosphate-buffered saline (PBS), or 3.3 × 10^4^ CFU P. aeruginosa M2 in the absence of burn. Mice were observed for clinical signs of sepsis and distress. Only mice that received a 10% TBSA burn and were infected with live P. aeruginosa M2 became moribund, with the onset of clinical symptoms occurring between 18 and 26 h and a mean time to death of 23 h. Mice in the other groups showed no symptoms, and all survived and continued to gain weight through the end of the experiment at 7 days postburn (Fig. 2A). Additionally, mice that received a burn alone gained weight over the course of 7 days after the procedure (data not shown). Another group of mice were burned and infected with 3.3 × 10^4^ CFU P. aeruginosa M2. Mice were euthanized at regular intervals between 0 and 26 h postburn (hpb). Bacterial burden was determined based on organ weight and blood volume. P. aeruginosa M2 was present in the skin directly after the burn and grew to >10^8^ CFU per gram of skin prior to spreading systemically at 18 hpb. P. aeruginosa M2 was not detected in the blood or organs prior to the onset of clinical symptoms at 18 hpb and rapidly increased in number at these sites thereafter until death (Fig. 2B). P. aeruginosa M2 was detected postburn only in mice that received s.c. infection. No other organisms were detected in any of our experiments.

### Inflammatory and anti-inflammatory cytokine levels in the serum in response to burn and infection with P. aeruginosa.

**FIG 3:**
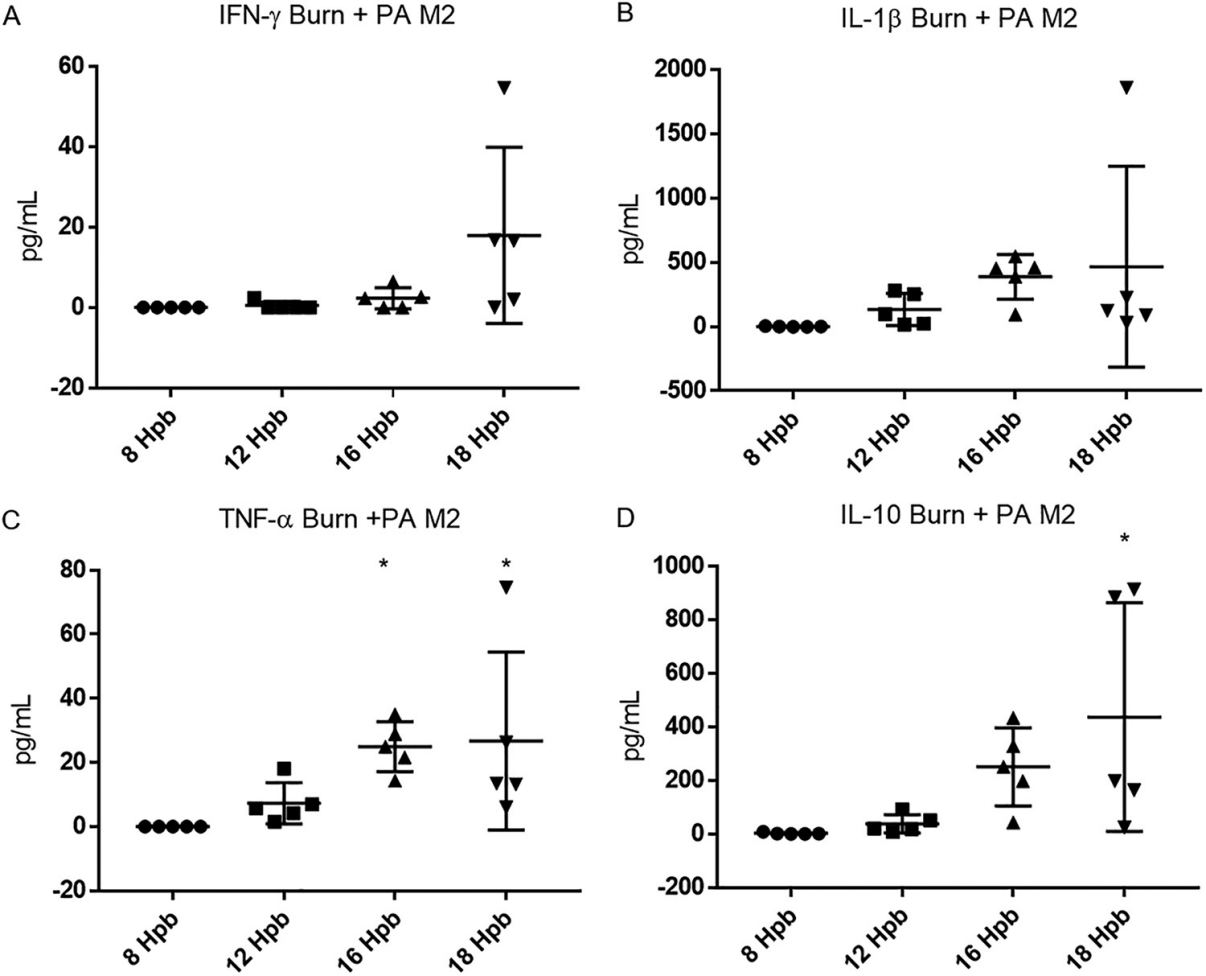
Serum cytokine concentrations postburn. Whole blood was collected via cardiac puncture at indicated times after burn and immediate P. aeruginosa M2 infection and cytokine concentrations were determined. IFN-γ (A), IL-1β (B), TNF-α (C), and IL-10 (D) levels for burn with infection were examined. One-way ANOVA was used to analyze serum cytokine concentrations to 8 hpb. *, *P* < 0.05; **, *P* < 0.01; ***, *P* < 0.001; ****, *P* < 0.0001; compared to sham or 8 hpb.

**FIG 4:**
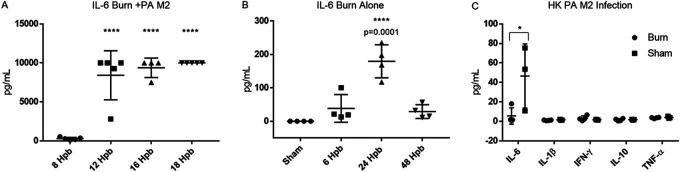
Serum IL-6 concentrations. Whole blood was collected via cardiac puncture at indicated times with described treatments. (A to C) Burn alone (A); burn with P. aeruginosa M2 infection (B); burn with HK P. aeruginosa M2 injection (C). One-way ANOVA was used to analyze serum cytokine concentrations. *, *P* < 0.05; **, *P* < 0.01; ***, *P* < 0.001; ****, *P* < 0.0001; compared to sham or 8 hpb.

We performed serum cytokine analyses to determine the global inflammatory response to burn alone and burn with infection with live P. aeruginosa M2, heat-killed (HK)-PA M2, or injection of P. aeruginosa M2 LPS. In burned mice that received an infection with live P. aeruginosa M2, proinflammatory cytokines gamma interferon (IFN-γ), interleukin-1 beta (IL-1β), and tumor necrosis factor alpha (TNF-α) became elevated at the onset of clinical symptoms (12 to 18 h) and remained elevated until the mice required euthanasia (Fig. 3A to C). IL-10, an anti-inflammatory cytokine, was also elevated along with the inflammatory cytokines and persisted until euthanasia (Fig. 3D). IL-6 levels were higher than the range of detection in burned mice with infection by 12 hpb (Fig. 4A). In burned mice that did not receive an infection, IL-6 increased at 24 hpb to over 200 pg/ml and returned to undetectable levels by 48 hpb (Fig. 4B). Additionally, IL-6 was increased when burned and sham mice were injected with a comparable amount of HK P. aeruginosa M2. However, the concentration of serum IL-6 in burned mice was lower than that of sham mice after injection with HK P. aeruginosa M2 (Fig. 4C). No cytokine response was observed after injection of 10 pg of P. aeruginosa M2 LPS in either burned or sham mice (data not shown). We assessed the concentration of activated transforming growth factor beta (TGF-β), a cytokine involved in tissue repair and immune suppression (14, 15), in the serum of mice that were burned, without and with an infection, as well as in mice that were infected in the absence of a burn. Although the level of total TGF-β was elevated, we saw no alterations in the activated fraction of TGF-β at any time point postburn without and with infection (data not shown).

### Increased susceptibility to s.c. P. aeruginosa infection at the burn site wanes after 72 h and causes increased susceptibility at distal sites.

**FIG 5:**
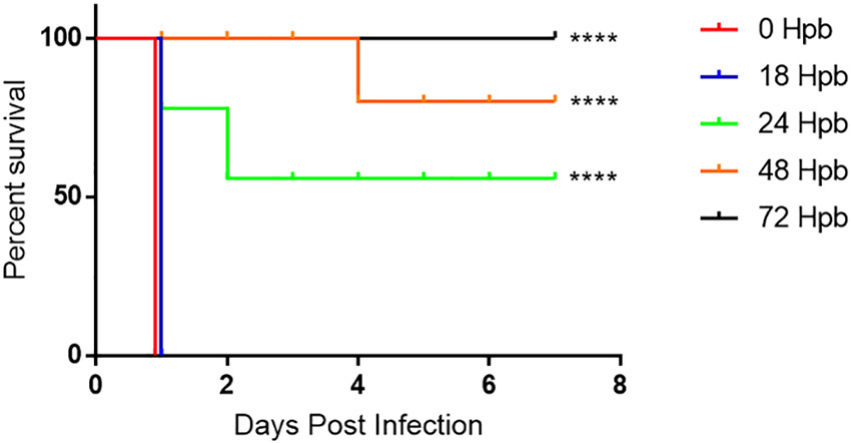
Increased susceptibility to P. aeruginosa M2 wanes over time after burn. Mice were infected s.c. with 600 CFU P. aeruginosa M2 at the burn site at 0 (*n* = 10), 18 (*n* = 10), 24 (*n* = 9), 48 (*n* = 10), or 72 (*n* = 10) hpb. Mice were observed for 7 days and euthanized upon the onset of morbidity. A log-rank (Mantel-Cox) test was used to compare survival between groups. ****, *P < *0.0001.

We observed that unburned sham mice were relatively resistant to s.c. P. aeruginosa M2 infection (50% lethal dose [LD_50_], ∼10^6^ CFU) (Table 1). In contrast, when burned mice were challenged s.c. with 600 CFU of P. aeruginosa M2 immediately following the burn and up to 18 hpb, the infection was 100% lethal. When bacterial challenge occurred at 24 and 48 hpb, the survival rate increased to 55% and 80%, respectively. By 72 hpb, mice were no longer susceptible to sublethal challenge (Fig. 5). This suggests a rapid, short-lived impairment in the host immune response to the burn. We also infected burned mice via routes that were distinct from the burn (i.e., intravenous [i.v.], intraperitoneal [i.p.], and skin outside the burn borders [s.c., nonburn]) immediately following the burn. We observed a 2-log reduction in the LD_50_ for i.v. and i.p. routes of infections as well as a 1-log reduction in the LD_50_ for an s.c. infection of nonburned skin outside the burn wound relative to sham mice. There was also a noticeable difference in the time of onset of clinical symptoms and morbidity among all groups (Table 1).

**TABLE 1:** Reduction in P. aeruginosa M2 LD_50_ at different sites when given directly after burn^*a*^

Route of infection at time of burn	LD_50_		Onset of morbidity (h)
	Sham	Burned	
Subcutaneous (at burn site)	10^6^	<600	18
Intravenous	10^6^	10^4^	48–72
Intraperitoneal	10^6^	10^4^	24−48
Subcutaneous (nonburn site)	10^6^	10^5^	48–72

a Mice were infected with various concentrations of P. aeruginosa M2 immediately postburn via s.c., i.v., and i.p. routes and observed for the onset of morbidity. Mice were euthanized when they reached a clinical score of 1 out of 3 (see Materials and Methods).

### Serum HMGB1 levels increase postburn regardless of P. aeruginosa infection.

**FIG 6:**
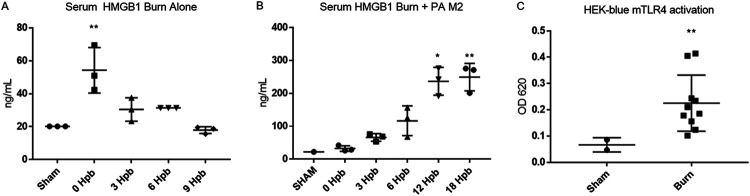
Serum HMGB1 levels and TLR4 activation postburn. Groups of 4 to 5 mice were burned and either left uninfected or infected with P. aeruginosa M2. Terminal blood samples were then collected from mice at the indicated times postburn. (A and B) Serum HMGB1 levels were measured with and without P. aeruginosa M2 infection. One-way ANOVA was used to compare burned and sham groups. Note that the *y* axis of panel B is much greater than that of panel A due to concentrations. (C) Serum taken from mice directly after burn without infection showed biological TLR4 activity. Student's *t* test with Welch’s correction was used to compare burned and sham groups. *, *P* < 0.05; **, *P* < 0.01; ***, *P* < 0.001; ****, *P* < 0.0001.

We next assessed the levels of serum HMGB1 in the context of burn injury and P. aeruginosa M2 infection. In burned, noninfected mice, HMGB1 levels rose to over 50 ng/ml immediately following the burn but returned to a baseline of ∼20 ng/ml after 9 hpb (Fig. 6A). In contrast, in the presence of infection, serum HMGB1 levels peaked at 250 ng/ml (i.e., >10-fold increase) after the burn with infection and remained elevated until death (Fig. 6B). In mice subjected to burn alone or burn with infection, increased HMGB1 concentrations preceded the elevation in serum cytokines. Due to the dramatic postburn increase in HMGB1, a TLR4 agonist (16), we employed an HEK-Blue mTLR4-driven NF-κB reporter cell assay to determine if the serum from burned mice at 0 hpb was biologically active and capable of stimulating the TLR4-mediated NF-κB stimulation. We observed that sera from burned mice increased stimulation of NF-κB activity in this assay, whereas negligible reporter activity was observed with sham mouse sera (Fig. 6C). We did not detect endotoxin in any of the sera by *Limulus* amoebocyte lysate (LAL) assay.

### *In vivo* inhibition of HMGB1-mediated TLR4 signaling using P5779.

**FIG 7:**
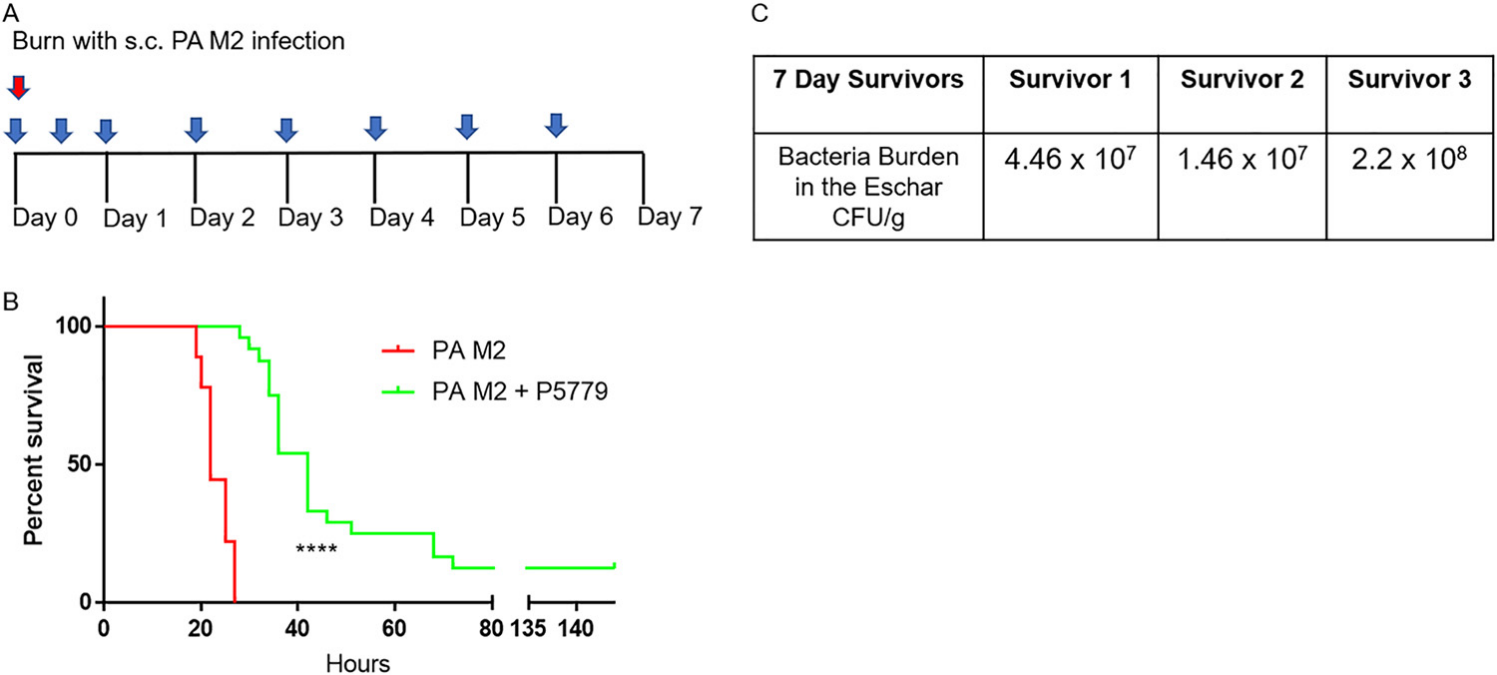
P5779 inhibition of HMGB1-mediated TLR4 signaling increased mean time to death and survival postburn with s.c. infection with P. aeruginosa M2. (A) All mice received a burn and were infected s.c. with ∼800 CFU P. aeruginosa M2 (red arrow). Control mice were observed for clinical signs of morbidity and infection. Treated mice were administered 500 μg of P5779 i.p. immediately following the burn, at 12 h, and once daily for 6 days (blue arrow). (B) Kaplan-Meier survival plot of control group (*n* = 10) and treated (*n* = 24) mice. Control mice had a mean time of death of 23 h, while treated mice had a mean time of death of 42 h. Survival, 0/10 control mice and 3/24 P5779-treated mice. A log-rank (Mantel-Cox) test was used to compare survival between groups, *P* < 0.0001. (C) Bacterial burden of surviving mice at 7 days postburn plus P. aeruginosa M2 plus P5779.

To determine if the observed HMGB1 levels were linked to mortality, we treated mice with P5779, a small-molecule inhibitor of TLR4 stimulation via HMGB1. P5779 specifically acts by blocking the MD-2-HMGB1 interaction without interfering with the binding of LPS to MD-2 (17). All mice received a burn and an s.c. infection with P. aeruginosa M2. The treated mice were administered P5779 immediately following the burn, at 12 hpb, and every 24 h for 6 days (Fig. 7A). Starting at 18 hpb mice were observed for clinical signs of sepsis every hour. All 10 control mice required euthanasia by 26 h with a mean survival of 23 h. In contrast, P5779-treated mice did not begin to succumb until 26 hpb and had a mean survival of 42 h. Three mice survived to 7 days and were not included in calculating mean time of death (Fig. 7B). After 72 h, surviving mice showed no signs of clinical disease or distress. The 3 mice that survived to 7 days were euthanized, and bacterial burden revealed an average of 9.60 × 10^7^ CFU/g of tissue in the eschar, while the skin and spleen had no detectible bacteria (Fig. 7C).

## DISCUSSION

The flame-based burn procedure employed here resulted in a nonlethal, full-thickness burn with the cellular destruction confined to the epidermis and dermis (Fig. 1). These findings were comparable to the burn depths achieved, visualized by H&E-stained slides of burned and sham skin by Leung et al. in their scald burn study in which they achieved partial and full-thickness scald burns (9). Directly after the burn, mice were injected with 3.3 × 10^4^ CFU of live P. aeruginosa M2 (two logs lower than the s.c. LD_50_ for sham mice) or equivalent amounts of HK P. aeruginosa M2 or purified P. aeruginosa M2 LPS s.c. at the burn site. Only mice that received an injection of viable P. aeruginosa M2 showed clinical symptoms or had detectable P. aeruginosa at the burn site or distal organs. Furthermore, all mice that were infected with viable P. aeruginosa M2 became moribund within 26 hpb. However, mice that received HK bacteria, LPS (from the challenge P. aeruginosa), or PBS postburn showed no clinical symptoms or any signs of distress, suggesting that the burn itself is not fatal but rather replicating P. aeruginosa M2 is required to cause disease (Fig. 2A). Mice that received an infection with 1 × 10^4^ CFU of live P. aeruginosa M2 without a burn remained in good health and gained weight. P. aeruginosa M2 could be isolated from the burn site directly after the infection and quickly increased in concentration. However, we did not detect P. aeruginosa M2 in the blood or peripheral organs prior to the onset of clinical symptoms. Once P. aeruginosa M2 was detectable in the blood and organs, the health of the mice rapidly deteriorated until they required euthanasia. P. aeruginosa M2 may need to replicate to a sufficiently high concentration in the skin to disseminate (Fig. 2B). Therefore, treatment to prevent P. aeruginosa M2 sepsis should begin prior to the bacterial dissemination from the skin. This continued presence of bacteria at the burn site provides the rationale for clinical targeting of the burn eschar.

Burned mice infected with P. aeruginosa M2 showed increased circulating proinflammatory cytokines (IL-6, IL-1β, TNF-α, and IFN-γ) and a rise in anti-inflammatory IL-10 late during infection once clinical symptoms were observed. These data are reminiscent of the cytokine response to Pseudomonas aeruginosa sepsis following cecal ligation and puncture in mice (18). Interestingly, it has been reported that a difference in the IL-10-to-TNF-α ratio was seen in patients with severe sepsis, with nonsurvivors having a ratio of >4.5 48 h after admission (19). The IL-10/TNF-α ratio in our study at 12 hpb with infection was 6.6 ± 2.4 and rose to 11.1 ± 3.7 at the onset of symptoms. Future work is needed to determine if this ratio can be used as a predictor of survivability of mice in the burn model.

In contrast, the only cytokine detected in mice that received a burn without infection was IL-6, which peaked at 24 h and returned to baseline levels after 48 h. The half-life of IL-6 in mice is between 8 and 12 h, suggesting that the IL-6 response to the burn occurred rapidly but did not persist in the absence of a secondary insult, such as a superimposed bacterial infection (20). Most interestingly, there was a difference in serum IL-6 levels between burned and sham mice that received an injection of HK P. aeruginosa M2. At 3 hpb, sham mice produced >200% more serum IL-6 on average than mice with burn injury. This suggests that viable tissue at the injection site is required for an initial robust IL-6 response (Fig. 4), which has been observed in a mouse model of turpentine-induced tissue inflammation l (21). Based on these cytokine data, we conclude that the flame burn produces an initial short-lived IL-6 response from the viable tissue adjacent to the burn site that is transient in the absence of an infection.

We confirmed previous data showing reduction of LD_50_ at distal sites that are outside the area of cellular destruction of the burn and the waning of the increased susceptibility to P. aeruginosa M2 infection at the burn site after 72 h (Table 1 and Fig. 5) (11). Together, along with the transient nature of IL-6 postburn, this suggests that there must be other soluble factors generated from the burned tissue that influence the general host defenses very soon after tissue destruction from the flame.

High-mobility group box 1 protein (HMGB1) is passively released from necrotic cells and increases in the circulation directly after burns in both humans and rats (13, 22, 23). HMGB1 is also actively released in low concentrations by responding innate immune cells, such as macrophages, early during bacterial infection. In the later stages of sepsis, hepatocytes and other somatic cells actively release HMGB1 in very high concentrations (24). It is likely that the massive immediate tissue destruction from the burn passively releases HMGB1 into the circulation, which caused activation of NF-κB through the TLR4-MD-2 signaling pathway. We screened serum HMGB1 levels postburn, without and with infection. After a burn without infection, serum HMGB1 levels rose significantly to 50 ng/ml within minutes of the burn and returned to baseline levels within 9 h. In contrast, burned mice that received a lethal s.c. infection with P. aeruginosa M2 exhibited serum HMGB1 levels that increased immediately after the burn and continued to increase to a concentration of >250 ng/ml at the time of morbidity. We saw an increase in TLR4-mediated NF-κB stimulation *in vitro* with serum from burned, noninfected mice compared to sham mice, suggesting that some factor(s) in the sera can biologically activate NF-κB through the TLR4 pathway after the burn and may be responsible, in part, for the observed cytokine cascade. Samples were screened for endotoxin by LAL assay. No endotoxin was detected in the serum that was used to stimulate the NF-κB reporter cell line; however, serum that was drawn at 18 hpb with infection contained approximately 0.24 ng/ml endotoxin. Disulfide-containing HMGB1 signals through the TLR4/MD2 pathway cause inflammation via NF-κB activation (17, 25). There are three HMGB1 redox isoforms (all-thio, disulfide, and oxidized), each with its own function (26). Only the disulfide-linked isoform is an active TLR4 agonist (27). Future studies should determine the isoform proportions and if the HMGB1 is passively or actively released after the burn with infection with P. aeruginosa M2 (Fig. 6).

P5779 is a small-molecule antagonist of HMGB1 that acts by inhibiting the association between HMGB1 and MD 2, which prevents HMGB1-mediated TLR4 signaling (28). P5779 has been used to reduce mortality in mice in other inflammatory-related diseases, such as influenza (29). To assess if circulating HMGB1 postburn affects mortality, burned and infected mice were treated i.p. with P5779 (Fig. 7A). Treatment of burned and infected mice with P5779 significantly prolonged survival, with 3 of 24 (12.5%) surviving the length of the experiment, to 7 days (Fig. 7B). At 72 hpb, the remaining three P5779-treated mice showed no signs of disease. The bacterial burden in the surviving mice was high in the eschar despite the lack of clinical symptoms. No bacteria were detected in the blood or spleen of the surviving mice. The survival of these mice and their lack of clinical symptoms after 72 h are concurrent with the return of normal host defenses at 72 hpb. The increase in survival in the P5779-treated group shows that circulating HMGB1 contributes significantly to the inflammation generated from the burn plus infection and suggests that HMGB1 should be studied further as a potential therapeutic target. Although we saw some survival and an increased mean time to death, other factors may be involved in P. aeruginosa M2-related mortality postburn. HMGB1 is only one of many DAMPs: heat shock proteins, HSP100, mitochondrial DNA, extracellular DNA and RNA, and others are released after tissue trauma and signal through multiple pattern recognition receptors (30). It is possible that other DAMPs contribute to morbidity and mortality in this flame-based mouse burn infection model and should be addressed in future studies.

Most laboratory-based burn research studies use scalding water baths or heated brass rods to achieve a burn (31). These models mimic the effects from scalds and touching hot surfaces but may not be optimal for understanding the effects of a flame burn, as heat may transfer into the animal differently and result in different secondary and tertiary effects. There are relatively few studies of flame burn models in mice. Burn size and final subdermal temperatures may dictate the inflammatory response and susceptibility or even mortality from the burn alone. Our 10% TBSA flame burn resulted in a nonlethal burn that increased susceptibility to P. aeruginosa M2 infections. The rate at which our mice became moribund from infection with P. aeruginosa is comparable to P. aeruginosa infections in mice that received a nonlethal 6% to 8% TBSA full-thickness scald burn (32). The lethality of the burn without infection is most likely due to the total size of the burn. Larger (18%) surface area burns in mice were shown to have mortality rates of 22% for scald burns and 11% for flame burns in the absence of infection (33). Our flame-resistant template produced a highly reproducible 10% TBSA burn when using Meeh’s constant of 9.74 for CD1 mice to calculate burn area (34). Our immune responses differ from results found in C57BL/6 mice that received a 35% TBSA scald burn (35). These studies found increased IL-1β and TNF-α postburn in the absence of infection within the first 24 h. This could be due to the larger percentage of the burn size or possibly from the heat transfer of the scalding water. The initial subdermal temperature of a 90°C scald burn and an ethanol-based flame burn are similar. However, after 9 s, the subdermal temperature from a scald burn can be 19°C higher than a flame burn in mice (33).

Taken together, these data suggest that a nonlethal 10% TBSA flame burn increased the susceptibility of mice to mortality from s.c. P. aeruginosa M2 infection even though there was only local destruction of the epidermis and dermis. Unlike burn models that are lethal in the absence of infection, this nonlethal burn model allowed us to examine the independent effects of the burn on the host response and identify a critical role for HMGB1 in burn wound sepsis. Since blockade of HMGB1 signaling postburn increased survival in mice, a similar blockade of HMGB1 signaling in burn patients may allow additional time for further interventions.

## MATERIALS AND METHODS

### Ethics statement.

All animal work was performed at the University of Maryland School of Medicine, which complies with guidelines for animal care established by the U.S. Department of Agriculture Animal Welfare Act, U.S. Public Health Services policies, and U.S. federal law. All animal experiments were approved by the University of Maryland School of Medicine Institutional Animal Care and Use Committee.

### Burn procedure.

Burn wounds were administered as previously described (11). Briefly, 6- to 10-week-old female Crl:CD-1 mice (Charles River Laboratories, MA) between 28 and 34 g had the hair on their backs clipped 24 h prior to infection. On the day of infection, they were sedated with 5% isoflurane for 8 min. The burn induced by pressing a flame-resistant polymer card with a 2.5-cm by 4.0-cm cutout (representing ∼10% of total body surface area) on the clipped back, followed by the application of 0.5 ml of 200-proof ethanol spread uniformly into the cutout, ignited with a flame, and allowed to burn for precisely 10 s. Mice were infected directly after the burn by subcutaneous injection at the burn site with 100 μl of bacterial suspension (∼10^4^ CFU; a sublethal dose in sham-treated mice but a 3-log LD_50_ for burned mice) or PBS. Mice were administered 500 μl Ringer's solution given intraperitoneally. Mice were allowed to recover on an HTP-1500 veterinarian-grade heating blanket (Adroit Medical Systems, Loudon, TN). A 3-log LD_50_ challenge dose initiated onset of clinical symptoms at ∼18 hpb. Sham mice had their hair clipped and received the same amount of anesthesia as the experimental groups but neither burn nor infection. Mice were observed twice a day postburn for clinical signs of sepsis and distress. A score between 1 and 3 was given to each mouse, 3 being normal appearance with no signs of distress and 1 requiring euthanasia, based on a previously described scoring system (36).

### Burn pathology.

Following terminal anesthesia, cardiac puncture was performed to obtain blood. The skin was then dissected from the burn site as follows. The surgical area was cleaned with 70% ethanol. Tissue was removed with a scalpel and surgical scissors in a 1-cm-by-1-cm square that extended from the surface of the epidermis through the skeletal muscle. Samples were placed in cassettes and allowed to fix for 72 h in 10% formaldehyde prior to being sectioned and stained with H&E. Slides were examined by a blinded dermatologic pathologist and imaged at ×10, ×40, and ×60 magnification using a BZX all-in-one microscope system (Keyence, Itasca, IL).

### Bacterial preparation.

An inoculum of P. aeruginosa M2 (O5/FlaB), originally isolated from the gastrointestinal tract of a CF-1 mouse by Ian Alan Holder (37), was grown overnight to stationary phase from glycerol stocks in 2.0 ml of Hy-Soy broth, containing 0.5% sodium chloride (American Bio, Canton, MA), 0.5% HY-Yeast (Kerry Bio-Science, Norwich NY), and 0.25% animal-free soytone (Teknova, Hollister, CA). One-hundred twenty microliters of overnight stationary P. aeruginosa M2 was transferred into 12 ml of fresh medium and grown to mid-log phase (optical density at 600 nm [OD_600_] of 0.6 to 0.8). Bacteria were pelleted, washed, and resuspended (to an OD_600_ of 0.3, corresponding to ∼1 × 10^8^ CFU/ml) in sterile PBS. Final dilutions were made to achieve the desired concentrations and verified by colony count on TSA (tryptic soy agar; Sigma-Aldrich, St. Louis, MO) plates.

### HK Pseudomonas aeruginosa.

Log-phase Pseudomonas aeruginosa was heat killed at 80°C for 60 min in a water bath. Viability was determined by plating on TSA plates that were incubated for 24 h at 37°C.

### LPS purification and lipid A isolation.

P. aeruginosa LPS was isolated and purified using a hot phenol-water extraction method after growth in lysogenic broth (LB) supplemented with 1 mM MgCl_2_ at 37°C (38). Subsequently, LPS was treated with RNase A, DNase I, and proteinase K to remove contaminating nucleic acids and proteins (39). Individual LPS samples were additionally extracted to remove contaminating phospholipids (40) and TLR2-contaminating proteins (41). Finally, individual LPS preparations were resuspended in 500 μl of water, frozen on dry ice, and lyophilized. To confirm the structure of the lipid A component of the purified LPS, lipid A was isolated after hydrolysis in 1% SDS at pH 4.5, and purity was confirmed by mass spectrometry as described previously (42). Briefly, 500 μl of 1% SDS in 10 mM Na-acetate, pH 4.5, was added to a lyophilized sample. Samples were incubated at 100°C for 1 h, frozen, and lyophilized. The dried pellets were resuspended in 100 μl of water and 1 ml of acidified ethanol (100 μl 4N HCl in 20 ml 95% ethanol). Samples were centrifuged at 5,000 rpm for 5 min at 4°C. The lipid A pellet was further washed (3×) in 1 ml of 95% ethanol. The entire series of washes was repeated twice. Samples were resuspended in 500 μl of water, frozen on dry ice, and lyophilized.

### Cell culture conditions and stimulations.

HEK-Blue-mTLR4 cells were purchased from InvivoGen (San Diego, CA, USA). They are stably transfected with mouse TLR4 (mTLR4), myeloid differentiation factor-2 (MD-2), cluster of differentiation-14 (CD14), and an inducible embryonic alkaline phosphatase (SEAP) reporter gene. The cells were cultured and maintained at 37°C with 5% CO_2_ in complete Dulbecco’s modified Eagle medium (DMEM) (Gibco, NY) supplemented with 10% heat-inactivated fetal bovine serum (FBS) and 1× HEK-Blue selection medium (InvivoGen, San Diego, CA), an antibiotic mixture for maintenance of HEK-Blue mTLR4 cell lines. The cells were seeded at 2.5 × 10^4^ cells/well in a 96-well plate and stimulated for 24 h with 100 μl undiluted sera from sham and burned mice. Salmonella enterica serotype Minnesota R595 lipid A (lot number MLA-24A; List Biological Laboratories, Inc.) was used as a positive control. The activation of NF-κB in HEK-Blue mTLR4 cells in response to TLR4 agonists was determined by a SEAP reporter assay at a wavelength of 620 nm using a VERSA max microplate reader (Molecular Devices, San Jose, CA). Endotoxin levels were assessed by Endosafe PTS chromogenic *Limulus* amebocyte lysate assay cartridges (Charles River, MA).

### Bacterial burden.

Organs (skin from the burn site, liver, spleen, and blood) were harvested postterminal bleeding and cervical dislocation using an aseptic technique. One milliliter of sterile PBS was added to 0.2 g of tissue sample and mechanically homogenized with a Dounce homogenizer while on ice for 10 s. Ten microliters of 10-fold serial dilutions of homogenized tissues were plated on TSA and incubated at 37°C overnight. Colony counts were conducted to determine the number of CFU per gram of tissue.

### Terminal blood collections for bacterial counts, cytokine, and DAMP concentrations.

Mice were anesthetized with 5% isoflurane for 8 min and laid on their backs, and the chest was cleaned with 70% ethanol. A 25-gauge needle attached to a 1-ml syringe was inserted into the heart, and 0.5 to 1.0 ml of whole blood was collected in appropriate tubes (heparin, ETDA, or serum separation) depending on procedures.

### Cytokine levels.

IFN-γ, TNF-α, IL-1β, IL-6, IL-10, and TGF-β were measured in multiplex with a Luminex 100 reader in the Center for Innovative Biomedical Research (CIBR), Cytokine Core Laboratory, at the University of Maryland School of Medicine. All samples were run in duplicate and compared to internal controls.

### HMGB1 levels.

Serum HMGB1 was measured using a mouse-specific ELISA kit (cat. no. SEA399Mu; Cloud-Clone, Waltham, MA)

### *In vivo* inhibition of HMGB1 with P5779.

P5779 was resuspended in sterile PBS at a concentration of 5 mg/ml. Burned mice with s.c. infection of <800 CFU P. aeruginosa M2 were separated into two groups, control and treatment. The treated mice were administered 500 μg of P5779 i.p. immediately after the burn, at 12 hpb, and once daily for 6 days. Control mice, which received a 10% burn and s.c. infection with P. aeruginosa M2, were resuscitated with 500 μl of Ringer's solutions i.p. directly after the burn. Treated mice received 400 μl of Ringer's solution so as not to increase the volume of resuscitation fluid.

### Statistical analysis.

All statistics were performed on GraphPad Prism 7 (GraphPad Software La Jolla, CA). Log-rank test for trend was used to analyze Kaplan-Meier survival curves. One-way analysis of variance (ANOVA) test with Dunnett’s multiple-comparison test was used to analyze cytokine and DAMP responses between groups over time.
